# Samidorphan/olanzapine combination therapy for schizophrenia: Efficacy, tolerance and adverse outcomes of regimen, evidence-based review of clinical trials

**DOI:** 10.1016/j.amsu.2022.104115

**Published:** 2022-06-30

**Authors:** Syeda Tayyaba Rehan, Abdul Hannan Siddiqui, Zayeema Khan, Laiba Imran, Abdul Ahad Syed, Muhammad Junaid Tahir, Zahra Jassani, Manjeet Singh, Muhammad Sohaib Asghar, Ali Ahmed

**Affiliations:** a Department of Medicine, Dow University of Health Sciences, 74200, Karachi, Sindh, Pakistan; b Department of Medicine, Lahore General Hospital, 54000, Lahore, Punjab, Pakistan; c Department of Psychiatry, Liaquat National Hospital and Medical College, 74800, Karachi, Sindh, Pakistan; d Department of Internal Medicine, Liaquat National Hospital and Medical College, 74800, Karachi, Sindh, Pakistan; e Department of Medicine, Dow University of Health Sciences, Ojha Campus, 75300, Karachi, Sindh, Pakistan; f School of Pharmacy, Monash University, Jalan Lagoon Selatan, Bandar Sunway, 47500, Subang Jaya, Selangor, Malaysia

**Keywords:** Samidorphan, Olanzapine, Schizophrenia, Review, Research, Systematic, Psychiatric

## Abstract

**Introduction:**

Schizophrenia is a complex medical illness characterized by hallucinations, delusions, and cognitive issues. Olanzapine, a second-generation antipsychotic widely prescribed for schizophrenia has proven to be efficacious, however, its use is associated with major adverse effects such as weight gain, metabolic syndrome and diabetes mellitus. Recently, FDA approved a combination dose of olanzapine and samidorphan (OLZ/SAM) to mitigate the adverse outcomes associated with olanzapine use for the treatment of Schizophrenia.

**Objectives:**

The approval of olanzapine/samidorphan combination by FDA in treatment of schizophrenia and bipolar I disorder has been a milestone. This article summarizes the clinical trials reporting the clinical efficacy and adverse effects of olanzapine/samidorphan combination along with their bias assessment.

**Methods:**

Pubmed, science direct, Ovid SP and Google Scholar were comprehensively searched for data collection. Clinical trials reporting the efficacy and adverse outcomes of the OLZ/SAM regimen were included in the review and the Cochrane risk of bias assessment tool (RoB 2.0, version 2019) was used to assess the risk of bias in each study.

**Results:**

Five trials employed the use of Positive and Negative Syndrome Scales (PANSS) and Clinical Global Impression-Severity (CGI-S) scale to assess the efficacy of OLZ/SAM. Overall, OLZ/SAM showed a significant reduction in PANSS total scores and CGI-S scores and might be a viable option for long-term treatment. The safety of combined therapy is assessed by trials considering the factors of ECG parameters, suicidal events, and movement disorders. Major adverse events included nervous system disorders, changes in blood chemistry, and metabolic or nutritional disorders, with worsening of adverse outcomes observed in a total of nineteen cases in six studies.

**Conclusion:**

The FDA-approved drug recombination of OLZ/SAM for the treatment of schizophrenia revealed efficacious outcomes and was generally well tolerated by patients partaking in various trials. The potential of samidorphan in mimicking the efficacy of olanzapine while mitigating olanzapine-induced weight gain makes it a promising regimen for improving symptoms and health outcomes in schizophrenic patients.

## Highlights

• FDA approved a combination dose of olanzapine and samidorphan (OLZ/SAM) for treatment of schizophrenia and bipolar I disorder.
• Overall, OLZ/SAM showed a significant reduction in symptoms for long-term treatment.
• Major adverse events included nervous system disorders, blood, metabolic or nutritional changes.

## 1 Introduction

Schizophrenia is a complex long-term medical illness characterized by hallucinations, delusions, disorganized thinking, and cognitive issues. It affects almost 1% of the population but is a major contributor to the global burden of disease [1]. A study conducted by Charlson et al. estimated that 21 million people are living with schizophrenia worldwide and the majority of these individuals belong to low- and middle-income countries (LMIC) [2]. Individuals between 15 and 30 years of age are at a higher risk of developing schizophrenia [3]. Schizophrenics are unable to live independent lives and are often dependent on their family and loved ones due to their incapability to support themselves [3]. It has also been reported that 4.9% of patients suffering from schizophrenia will commit suicide during their lifetimes, near illness onset [3]. Suicide has been found to be a leading cause of decreased life expectancy in schizophrenia [4]. In a 2017 meta-analysis conducted by Hjorthøj et al. schizophrenia was associated with a weighted average of 14·5 years of potential life lost (95% CI 11·2–17·8), and was higher for men than women (15·9, 13·8–18·0 vs 13·6, 11·4–15·8) [5]. It was also noted that life expectancy was lowest in Asian and African regions [5]. The World Health Organization (WHO) estimated that direct costs of the disease in Western countries range from 1.6 to 2.6% of total healthcare expenditures [6]. Risk factors for schizophrenia include obstetric complications, time of birth, behavioral differences, and exposure to substance abuse [3]. Early on-set schizophrenia is commonly presented during early adulthood (late teens to early twenties) and associated predominantly with the male gender, a positive family history of Schizophrenia and other psychiatric conditions, particularly affective disorders [7]. Individuals born at the end of winter and beginning of spring are likely to develop schizophrenia later in life due to there being a higher chance of influenza virus during winter [3]. Viral infections during pregnancy may lead to changes in the brain leading to schizophrenia [3].

The disease progression for schizophrenia has been unclear which has hindered the development of novel treatments for alleviating the symptoms of schizophrenia but dopaminergic hypothesis is a widely accepted one due to evidence of the efficacy of antipsychotics to act on Dopamine (D2) receptors [8].

### 1.1 Current treatments for schizophrenia

At present, antipsychotics are the mainstay treatment for schizophrenia. First-generation antipsychotics’ main mechanism of action is to block D2 receptors in the striatum [9]. Chlorpromazine was the first neuroleptic synthesized and is categorized as a first-generation antipsychotic belonging to the family of phenothiazines [9]. Phenothiazines are divided into three groups based on their aliphatic side chain which are aliphatic, piperidine, and piperazine phenothiazines [9]. Their non-selective nature leads to side effects like recurring extrapyramidal symptoms such as dyskinesia, dystonia, akathisia, unwanted movements, and increased prolactin hormone [9].

In more recent years, second-generation antipsychotics are prescribed to relieve the positive symptoms of hallucination, delusion but negative symptoms remain untreated which is a major drawback since 40% of patients with chronic schizophrenia display negative symptoms such as depression, dementia, chronic pain, etc [10]. The use of atypical second-generation antipsychotics have been the mainstay for treating schizophrenia, such as clozapine, olanzapine, aripiprazole, and ziprasidone [10].

Clozapine is a widely popularised treatment for schizophrenic patients who are “treatment-resistant” but it is only capable of relieving the positive symptoms such as hallucination, delusion, etc. while negative symptoms are left untreated such as emotional empathy [1]. There is also evidence of endocrine and sedative side effects when being treated with neuroleptics, along with being a contributor to decreased life span due to pro-arrhythmia effects [1,11]. The adverse effects of antipsychotic medications range from relatively minor tolerability issues like mild sedation or dry mouth to constipation, akathisia, sexual dysfunction, acute dystonias, disfiguring, weight gain, tardive dyskinesia, myocarditis, and agranulocytosis. Some adverse effects have little short‐term clinical implications, for example, increased prolactin or serum lipid levels [12]. Second-generation antipsychotics have a higher ability to block serotonin 5HTA receptors as compared to dopamine D2 receptors [13]. Due to this mechanism of action, there is a lower occurrence of extrapyramidal symptoms [13].

Presently, clozapine, olanzapine, quetiapine, risperidone, paliperidone, ziprasidone, and molindone are widely approved atypical antipsychotics for treating schizophrenia [14]. Despite a lower risk of extrapyramidal symptoms after treatment using second-generation antipsychotics, there is also increasing evidence of weight gain, a metabolic syndrome that leads to diabetes mellitus [14]. This is due to the blockage of adrenergic, cholinergic, histaminergic receptors by psychoactive agents [14]. Histamine H1 receptors antagonism has increasingly been linked to obesity and weight gain in schizophrenics. Metabolic side effects are commonly linked to clozapine and olanzapine [14].

Third-generation antipsychotics differ from other neuroleptics owing to their different mechanism of action as they are partial D2 agonists rather than antagonists [1]. Presently, aripiprazole, brexpiprazole, and cariprazine are approved for treatment [1]. Aripiprazole has a favorable side-effect profile when compared with other antipsychotics but it still has certain limitations in the form of adverse effects, akathisia, weight gain, agitation, insomnia, anxiety, and headache [1,15]. The severity of weight gain and metabolic syndrome is far less than olanzapine. Treatment with aripiprazole leads to decreased serum prolactin levels [15].

### 1.2 Olanzapine chemistry

Olanzapine is a synthetic derivative of thienobenzodiazepine with antipsychotic, antinausea, and antiemetic activities [16]. It is a selective monoaminergic antagonist which binds with high affinity with serotonin, dopaminergic, muscarinic, histamine, and alpha-1 adrenergic receptors [16]. Olanzapine's exact mechanism of action as a therapeutic agent in schizophrenia is relatively unknown, but it is considered to act as an antagonist to dopamine D2 receptors along with rapid ligand-receptor dissociation kinetics which helps to decrease extrapyramidal symptoms [16]. Along with it being used as a treatment for schizophrenia, it is also used as a treatment for bipolar illnesses [16]. Olanzapine is a counterpart of clozapine which has similar pharmacological properties but it has fewer autonomic side effects and it is not associated with agranulocytosis which is very prevalent amongst other atypical second-generation antipsychotics [1].

### 1.3 Samidorphan chemistry

Samidorphan (SAM), 3-carboxamido-4-hydroxy naltrexone, is a relatively new opioid system modulator that has a high affinity for binding with μ‐opioid, κ‐opioid, and δ‐opioid receptors while acting as an antagonist at μ‐opioid receptors [17]. It acts as a partial agonist at k-opioid and δ‐opioid receptors. Functionally, in vivo, SAM primarily acts as an opioid receptor antagonist [17]. It is primarily eliminated through hepatic metabolism and renal excretion [18].

### 1.4 Olanzapine as a mono-therapy

Olanzapine is a widely prescribed antipsychotic for the treatment of schizophrenia as well as maintaining the symptoms of agitation in bipolar disorder. A long-acting injectable (LAI) is available to treat schizophrenia [19]. Olanzapine was approved in 1996 as a therapeutic agent for treating schizophrenia [19]. Olanzapine has been widely used to treat schizophrenia in first time episodes as evident in a study titled, Recovery After an Initial Schizophrenia Episode study's Early Treatment Program (RAISE-ETP), in which 300 participants were treated with olanzapine between July 2010 to July 2012 as an effective monotherapy and remained the second most prescribed antipsychotic (17%) after risperidone in the United States (US) [19].

A 2019 meta-analysis conducted by Kashimoto et al. included data from 59 studies and 45,787 participants which compared head-to-head efficacy of various antipsychotics [20]. Regarding all-cause discontinuation, clozapine, olanzapine, and risperidone were superior to several other second-generation antipsychotics. Similarly for relapse prevention, olanzapine was found to be superior to chlorpromazine and haloperidol [20].

Schizophrenia subjects are reported to have a higher frequency of smoking which affects the pharmacokinetics of olanzapine [21]. Around 60% of Schizophrenia patients are smokers [21]. CYP1A2 is inducible by polycyclic aromatic hydrocarbons (PAHs) present in cigarette smoke leading to increased clearance and decreased plasma levels of olanzapine [22]. Smokers have 53% increased clearance of olanzapine as compared to non-smokers [23]. The efficacy and safety of the olanzapine treatment are also affected by sex and age. Namely, women and older subjects responded better to therapy. They also displayed increased adverse effects such as relatively increased BMI and extrapyramidal symptoms as evident by Djordjevic et al.’s study [22].

### 1.5 Olanzapine's efficacy in other conditions

Evidence of olanzapine as an effective agent in treating mood disorders when used in combination with fluoxetine was reviewed by Luan et al. in a 2017 meta-analysis [24]. Five trials with 3,020 participants were studied in clinical trials which showed increased effectiveness in relieving depressive and psychotic symptoms as opposed to being used as a monotherapy [24]. The olanzapine/fluoxetine combination is FDA approved for bipolar depression [24]. Regarding treatment response rate, it was found that the Olanzapine/Fluoxetine combination had a higher response rate than olanzapine mono-therapy but a similar response rate as with fluoxetine. This combination treatment had side effects such as weight gain, increased appetite, somnolence, fatigue, peripheral edema, and sedation [24]. Olanzapine has been found to be an efficacious alternative to haloperidol in the treatment of delirium which is an acute neuropsychiatric disorder characterized by fluctuating levels of consciousness and impaired cognitive function [25]. In a systematic review conducted by Riviere et al., olanzapine was found to have an 82.4% reduction in symptoms of delirium similar to haloperidol's 87.5% [25]. Olanzapine and haloperidol began to take effect at low dosages with olanzapine having a faster rate of manifestation [25].

### 1.6 Adverse effects

Despite its FDA approval, olanzapine has been shown to have adverse side effects including weight gain, metabolic syndrome, dyslipidemia, and diabetes mellitus. Presently, growing evidence leads to the conclusion that olanzapine treatment can lead to severe cardio-metabolic disorders such as dyslipidemia which contributes towards decreased life spans in schizophrenic patients [26]. In a meta-analysis conducted by Rong Li et al., several findings were noted [26]. In the first published cases of olanzapine-associated hypertriglyceridemia, nine patients on olanzapine were followed for an average of 16 months and observed an increase in serum triglycerides (TG) from the baseline mean of 170 mg/dl (range 25–200 mg/dl) to the mean of 240 mg/dl (range 135–369 mg/dl), with five patients sustaining >50% increase in serum TG [26]. Further research associated olanzapine with a roughly five-time increased risk of developing hyperlipidemia as compared with no antipsychotic exposure [26]. Moreover, with each 1 mmol/l increase in TG, the risk of cardiovascular disease (CVD) increases by ≈ 12% and 37% in men and women, respectively [26].

Olanzapine has also been linked with a negative influence on Heart Rate Variability (HRV) and the autonomic nervous system [27]. This is due to its mechanism of action on cardiac cholinergic receptors which increases K+ delivery suppressing the vagal system. It also blocks alpha 1 adrenergic receptors leading to vasodilation as well as hypertension [27]. Olanzapine's continued use as a monotherapy has been linked with cardiomyopathy affecting the function of cardiac autonomous systems [27]. The renin-angiotensin system also comes into action as an increased level of catecholamines excites the sympathetic functions [27].

One of the most recurring adverse effects of olanzapine is weight gain and obesity which has been reiterated in several studies. A study conducted by Cheng et al. indicated that after six weeks of treatment with olanzapine (5–20 mg/d) in Chinese patients with bipolar I disorder, significantly more patients receiving olanzapine experienced a clinically relevant weight gain (CRW) (24.1% vs. 1.4%), as well as a significantly higher, mean weight increase (2.44 vs. 0.16 kg) compared to placebo [28]. In a study by Shen, Zhao, Rosenzweig-Lipson et al. olanzapine caused significantly more weight gain (average mean weight change: 4.04 kg [2.51; 5.57]) over a course of six weeks compared to placebo (p < 0.001). The random-effects model resulted in a significantly higher risk for CRW of 17.00 [2.38; 121.63] compared to placebo (p = 0.005) [29].

## 2 Olanzapine and samidorphan combination therapy approval by FDA

In view of the adverse effects of olanzapine which are evident from various studies, such as weight gain, obesity, dyslipidemia, diabetes mellitus, and metabolic syndrome; U.S. FDA approved a combination dose of OLZ/SAM (LYBALVI™) to mitigate the effects of weight gain by olanzapine for the treatment of schizophrenia and bipolar I disorder [[26], [27], [28], [29], [30]]. It received its first approval on May 28, 2021 in the USA for treating not only schizophrenia but also bipolar I disorder (including acute treatment of manic or mixed episodes as monotherapy and as an adjunct to lithium or valproate, and maintenance monotherapy treatment) in adults [30]. The safety of LYBALVI was evaluated in 1262 patients (18–67 years of age) diagnosed with schizophrenia in four double-blind, controlled studies and three long-term safety extension studies of up to 3 years of duration. This experience corresponds to approximately 910 person-years. In these studies, there were a total of 663 patients exposed to LYBALVI for at least 6 months and 386 patients for at least one year [[31], [32], [33],[39], [40], [41], [42]].

FDA's decision to approve the combined regimen of OLZ/SAM is corroborated by the results of various long-term studies studying the efficacy and safety of this regimen [31,32]. Promising results of this combined regimen have been noticed in studies by Potkin et al. and Correll et al., showing similar efficacious results of this combined regimen in a phase 3 study [31,32]. The combined therapy was observed to reduce the negative outcomes of schizophrenia and also curb the olanzapine-induced weight gain in schizophrenia patients [33].

**Fig. 1:**
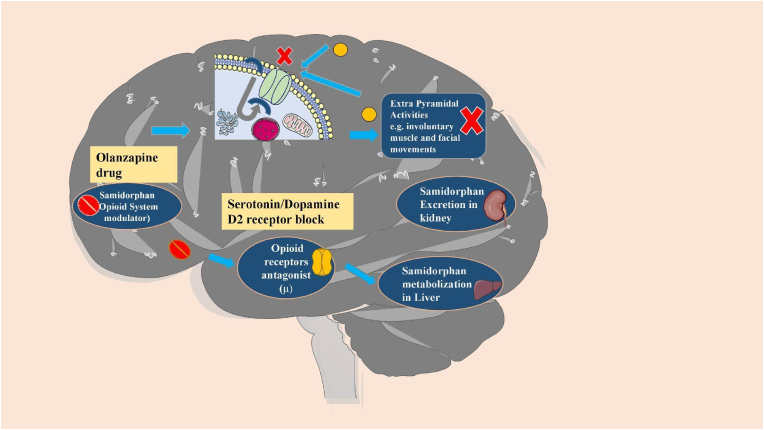
Pathomechanism of both the Olanzapine and Samidorphan drugs. Olanzapine blocks serotonin or dopamine D2 receptors to inhibit extra pyramidal activities. Samidorphan-an opioid receptor antagonist-gets metabolized in the liver and excreted through kidney in urine, contraindicated in patients with liver and kidney disease [[16], [17], [18]].

LYBALVI is a combination of olanzapine, an atypical antipsychotic, and samidorphan, an opioid antagonist (Fig. 1) [34]. This combination tablet is intended for oral usage and is available as a film-coated bilayer tablet in the following dose strengths: 5 mg/10 mg, 10 mg/10 mg, 15 mg/10 mg, and 20 mg/10 mg of olanzapine and samidorphan (equivalent to 13.6 mg of Samidorphan l-malate) [35]. Samidorphan has a bioavailability of 69% and a half-life of around 7–10 h while olanzapine has a half-life of 35–52 h which makes it a suitable companion to olanzapine when taken together as a combination tablet. Combining these two drugs together had no effects on either of their pharmacokinetics [34]. Following a single-administration of LYBALVI (10 mg olanzapine/10 mg samidorphan), the mean AUC_0-inf_ and C_max_ of olanzapine was 628 ng h/mL and 16 ng/mL, respectively. The mean AUC_0-inf_ and C_max_ of olanzapine after 10 mg single-dose administration of olanzapine tablet was 610 ng h/mL and 16 ng/mL, respectively [36]. After the 1 week, lead-in period of administration, the steady-state of AKLS 3831 was achieved approximately within a 1 week from 10 mg dose to a 20 mg dose (AKLS 3831 10/10 to AKLS 3831 20/20) [35].

OLZ/SAM carries a black box warning and is contraindicated in patients who are opioid users or patients who are undergoing an acute opioid withdrawal [37]. Samidorphan is an effective opioid antagonist and its use in opioid users can precipitate opioid withdrawal in patients who are dependent on opioids, which can lead to an opioid withdrawal syndrome, sometimes requiring hospitalization [37]. Prior to initiating LYBALVI, there should be at least a 7-day opioid-free interval from the last use of short-acting opioids, and at least a 14-day opioid-free interval from the last use of long-acting opioids [37]. It is also not approved for treatment for patients with dementia-related psychosis, as elderly patients with dementia once treated with antipsychotics have a higher risk of death [30].

Samidorphan/olanzapine regimen has also been approved for the treatment of bipolar disorder-I (BD-I) as a maintenance therapy to alleviate manic and mixed episodes [35]. The approval of the combined treatment has been fueled by olanzapine monotherapy's efficacy in the treatment of BD-I [38]. Sun et al. demonstrated that olanzapine has no significant effects on the pharmacokinetics of lithium or valproate in BD-I treatment [39].

To the best of our knowledge, this is the first narrative review which has included 8 clinical trials conducted to gauge the efficacy of the combined therapeutic agent for alleviating the symptoms of schizophrenia. The purpose of reviewing the evidence-based clinical trials is to provide a comprehensive elucidation of the benefits and side effects of the intervention for clinicians to better understand its efficacy as a therapeutic agent. Numerous studies have associated olanzapine with various side effects including obesity, dyslipidemia, metabolic syndrome, diabetes mellitus and Heart Rate Variability (HRV) [26,27]. This new combined regimen although, superior to olanzapine monotherapy, is not without its adverse effects which have been weighed against its efficacy in alleviating the symptoms of schizophrenia.

## 3 Aims of the review

This narrative review aims to assess the efficacy, safety, and adverse outcomes of recently approved combination therapy of olanzapine and samidorphan for schizophrenia patients.

## 4 Methodology

A comprehensive search was performed on PubMed, Google Scholar, Science Direct and Ovid SP with the keywords “Samidorphan” AND “Olanzapine” OR ''LYBALVI” AND “Schizophrenia” by August 30th, 2021 (Appendix A, Appendix A).

## 5 Inclusion and exclusion criteria

All clinical trials providing efficacy (PANSS score, CGI-S score, weight changes), safety (ECG parameters, movement disorders, and suicides), and adverse outcomes of the OLZ/SAM combined regimen were included. All preclinical studies, case reports, case series, reviews, and clinical trials not providing the efficacy, safety, and adverse events of drugs in schizophrenia were excluded.

Data Extraction: Data was collected by two authors independently and a third author ratified the information. Baseline characteristics (phase of trial, registration number of trial, total participants in trial, OLZ/SAM doses administered, follow-up duration and aims of study) were extracted from shortlisted articles. Data was extracted for suicidal inclination of patients (successful, ideation and behavior), movement disorders observed in patients (dyskinesia, parkinsonism and akathisia), and the associated movement disorders assessment scales (AIMS, SAS, BARS). Severity of AEs was recorded as patients who experienced any, mild, moderate, severe, or discontinuation of drug adverse events. Along with that, patient data for nervous system disorders, metabolism and nutritional disorders, gastrointestinal disorders, changes in blood chemistry and any other serious AEs was also extracted.

Information regarding the efficacy (PANSS score, CGI-S score, weight changes), safety (ECG parameters, movement disabilities, suicides), and adverse outcomes of regimen were extracted from the selected clinical trials.

## 6 Risk of bias assessment

Two researchers (AHS, LI) used the revised Cochrane risk of bias assessment tool (RoB 2.0, version 2019, available at www.riskofbias.info) to conduct the test for authenticity and check the overall methods and outcomes of trials [38]. Any disputes were settled by a third researcher (STR). The updated version of the tool has five domains to check the biasness: (D1) randomization process; (D2) deviations from intended interventions; (D3) missing outcome data; (D4) measurement of the outcome; and (D5) selection of the reported results. For judgment, assessors were required to answer the various questions given in each domain. If the trial had any domain at a “high risk” of bias, we judged it to have a high risk of bias overall. Similarly, if a trial had “some concerns'' in one or more domains, it was judged to have some concerns overall.

## 7 Results

Our search in PubMed and Google Scholar yielded 3210 articles. After removing duplicate articles and abstract screening, 12 articles were shortlisted and 4 articles were further excluded after the full-text review. Of these 4 excluded articles, one trial was testing the regimen on healthy patients, while another trial only used the drug olanzapine. The remaining two were not clinical trials and consisted of patient exit interviews. We finally included 8 articles that were pivotal clinical trials. There were four-phase III, two-phase II, and two-phase I trials with a total of 2231 randomized patients. There is an ongoing phase III trial that will tell us the long-term safety, tolerability, and durability of treatment with OLZ/SAM in patients with schizophrenia.

## 8 Risk of bias

**Fig. 2:**
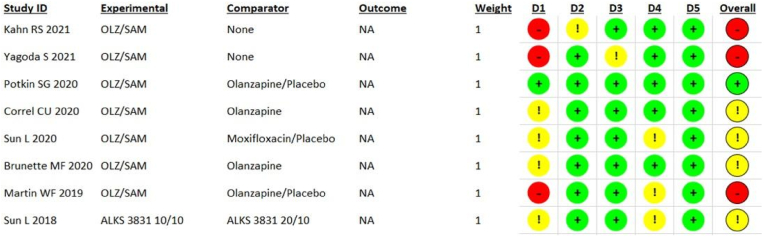
Risk of bias of included studies assessed on Cochrane risk of the bias assessment tool.

We used the revised Cochrane risk of the bias assessment tool and answered the questions for each domain using published journal articles. Trial protocols were also used when available (Table 1). All the analyses were done on an intention-to-treat model. The assessment highlighted 3 (37.5%) studies to have a high risk of bias overall (Fig. 2) [33,39,40]. While 4 (50%) studies had some concerns overall [32,36,41,42], and 1 (12.5%) study was assessed to be at low risk of bias overall [31]. A high risk of bias in the randomization process (D1) was the major reason for studies to be judged with a high risk of bias overall. Only one study had all domains that were assessed to be at low risk of bias [31].

**Table 1:** Demographic details of the recruited trials.

Trial	Phase	No of participants	Registration no.	Dosage (OLZ/SAM)	Follow up duration	Aim of study
**L. Sun et al. 2018**	I	42	NCT02804568	ALKS 3831 10/10 mg, 20/10 mg.	3 weeks.	The aim was to provide the PK profile of the active components of ALKS 3831, Olanzapine and Samidorphan, in patients of Schizophrenia.
**W.F. Martin et al. 2019**	II	347	NCT01903837	5/5 mg, 10/10 mg, 20/20 mg.	4 weeks.	The objective was to evaluate the safety, tolerability and efficacy of OLZ/SAM combined in Schizophrenia subjects.
**C.U. Correll et al. 2020**	III	550	NCT02694328	10/10 mg, 20/10 mg.	24 weeks.	The aim was to assess the weight profile of combined Olanzapine/Samidorphan compared with olanzapine in patients with Schizophrenia.
**S.G. Potkin et al. 2020**	III	401	NCT02634346	10/10 mg, 20/10 mg.	4 weeks.	The objective was to evaluate the antipsychotic efficacy of OLZ/SAM relative to Placebo in patients with acute worsening of Schizophrenia.
**L. Sun et al. 2020**	I	100	–	10/10 mg on days 2–4, 20/20 mg on days 5–8, 30/30 mg on days 9–13.	2 weeks.	The aim was to assess the effects of therapeutic and supratherapeutic doses of OLZ/SAM on cardiac repolarization in patients with Schizophrenia.
**M.F. Brunette et al. 2020**	II	229	NCT02161718	10/10 mg.	36–60 weeks. Follow-up for drug dispensing every 2 weeks, clinical and safety follow-up every 4 weeks.	The assessment of safety, efficacy, and tolerability of OLZ/SAM, administered as 2 tablets, compared with Olanzapine and matched Placebo tablets.
**R.S. Kahn et al. 2021**	III	265	NCT02694328	10/10 mg, 15/10 mg, 20/10 mg.	52 weeks.	Assessment of long-term safety and tolerability of OLZ/SAM in Schizophrenia patients.
**S.Yagoda et al. 2021**	III	277	NCT02634346	10/10 mg, 15/10 mg, 20/10 mg.	4 weeks.	The aim was to observe the long-term safety and tolerability of OLZ/SAM in adults with Schizophrenia
**Ongoing (Estimated completion date; December 2023)**	III	500	NCT03201757	–	–	Assess the long term safety, tolerability, and durability of treatment effect with ALKS 3831 in subjects with Schizophrenia.

PK= Pharmacokinetic.

ALKS 3831 = ALKS 3831 is a drug composed of samidorphan, and olanzapine, in a single bilayer tablet.

OLZ/SAM = olanzapine/samidorphan.

## 9 Efficacy of OLZ/SAM

Five trials employed the use of Positive and Negative Syndrome Scales (PANSS) and Clinical Global Impression-Severity (CGI-S) scale to assess the efficacy of OLZ/SAM. Overall, OLZ/SAM showed a significant reduction in PANSS total scores and CGI-S scores and can be considered for long-term treatment [31]. In a 52-week long phase III clinical trial OLZ/SAM was administered daily at doses of 10 mg/10 mg (10/10), 15 mg/10 mg (15/10), or 20 mg/10 mg (20/10). This combined regimen reduced the mean (SD) PANSS scores from 59.0 (11.8) at baseline to 58.3 (12.1), with a mean (SD) change of −0.2 (8.5) [41]. The CGI-S score was mean (SD) 3.1 (0.7) at baseline and had a mean change of −0.11 points by the end of the study [41]. Another 52-week long phase III clinical trial used 10/10, 15/10, and 20/10 mg OLZ/SAM does. This regimen resulted in a mean (95% Cl) change of −16.2 (−18.5 to −14.0) in PANSS total score, which was mean (SD) 78.9 (16.5) at baseline. CGI-S scores improved with a mean (95% CI) change of −0.9 (−1.0 to −0.8). This CGI-S score at baseline was mean (SD) 3.9 (1.0) [34]. A similar trend was observed in the 4-week long phase III trial (OLZ/SAM doses 10/10 and 20/10) in which baseline PANSS total score for patients in the OLZ/SAM group was mean (SD) 101.8 (11.6) and at week 4, it decreased by mean (SD) −23.7 (12.6). The CGI-S score at baseline for the same group was mean (SD) 5.1 (0.7) and the change by week 4 was mean (SD) −1.2 (0.9) [31]. A 24-week long phase III trial with the OLZ/SAM dose of 10/10 mg and 20/10 mg highlighted a least-squares mean (LSM) change of −8.2 (SE = 0.73) in PANSS total score [31], while the 12-week phase II trial (OLZ/SAM dose 5/5, 10/10, 20/20) reported LSM (95% Cl) change of −2.2 (−3.2 to −1.3) in PANSS total score [33]. Both of these trials reported a significant decrease in CGI-S scores of patients receiving the OLZ/SAM therapy [32,33]. Finally in the phase II trial reported by Brunette et al. [43] there was an overall improvement in PANSS total score and CGI-S score in the OLZ/SAM group by week 63, shown by LSM change of −5.4 (SE = 1.01) and −0.68 (SE = 0.11) respectively [43].

## 10 OLZ/SAM effects on body weight

Regarding weight change, Kahn et al. [41] reported no significant differences in OLZ/SAM and olanzapine monotherapy groups. The percentage of patients with significant weight gain or loss was similar in both groups. Martin et al. [33] explained the proportion of weight change to be lowered by 37% (absolute change = 1.9 kg) in the OLZ/SAM group versus olanzapine plus placebo group. The authors further explained the efficacy of OLZ/SAM by calculating that patients had a 2.7 times greater risk of gaining >10% of their body weight from baseline in the olanzapine plus placebo group than the OLZ/SAM group (odds ratio = 2.73, 95% CI = 1.11, 6.67, p = 0.023). Another trial by Correll et al. [32] confirmed the same outcomes by elucidating the stability of weight changes in the OLZ/SAM group from 6 weeks onwards, whereas weight kept on increasing in the olanzapine monotherapy group.

## 11 Safety of OLZ/SAM

The safety of OLZ/SAM therapy in schizophrenia patients was assessed in six trials. Three studies used various ECG parameters to evaluate the tolerance of this regimen in schizophrenia patients [41,42,44]. Five trials employed the use of the Columbia-Suicide Severity Rating Scale (C-SSRS) to assess suicidal ideation and suicidal behavior in patients [[31], [32], [33],^41^,^42^]. Parkinsonism, dyskinesia, and akathisia were evaluated as safety factors by three trials [31,41,42]. Overall OLZ/SAM's safety was well established and the patients showed tolerability to the drug regimen.

## 12 ECG parameters

In a 4-week long phase I trial, there were no noticeable effects on QTc interval of patients upon administering the combined regimen of OLZ/SAM up to supratherapeutic doses of both drugs. Mean QTcF were small (OLZ/SAM doses of 10/10, 20/20, and 30/30) ranging from −2.6 to 4.6 msec. The mean QTcF was below 5 msec at all time points for all OLZ/SAM doses. However on day 13, 4 h after administering OLZ/SAM 30/30 mg, there was a large increase in mean QTcF (4.6 msec; 90% CI: −2.08 to 11.28). The primary analysis was done for OLZ/SAM (10/10 to 30/30) using the C-QTc modeling, which excluded a clinically concerning QTc effect (ie, QTcF ≥10 msec). The values of PR and QRS were small and similar across the different OLZ/SAM dose levels, and the largest PR change was −8.9 msec. The mean QRS values were within ±2.0 msec for all time points after dose administration. There was a higher occurrence of flat and biphasic T-waves at the OLZ/SAM 20/20 and 30/30 mg dose levels. On day 8, flat T-wave was exhibited by 9 (16.7%) patients, while 5 (9.3%) patients exhibited a biphasic T-wave upon treatment with OLZ/SAM 20/20 mg. Treatment with OLZ/SAM 30/30 mg on day 13 resulted in 8 (15.4%) patients exhibiting a flat T-wave and 5 (9.6%) patients showing a biphasic T-wave [44]. Only 1 patient had an abnormal T wave on ECG in the phase III trial reported by Kahn et al. [41]. Yagoda et al. reported an abnormal post-baseline QT interval value in one patient [44].

## 13 Suicides

No suicides were reported in OLZ/SAM recipients by any of the included trials (Table 2). According to the C-SSRS, 6.8% of the patients in a trial by Kahn et al. [41] and 1.8% of patients in a study by Yagoda et al. [34] dealt with suicidal ideation during the dose administration period. Only 1 (0.4%) patient was recorded with suicidal behavior in both trials. Three trials reported no suicidal ideation or noticeable suicidal behavior in any of the patients receiving OLZ/SAM therapy [[31], [32], [33]].

**Table 2:** Representation of suicide, movement disorders, and movement disorder assessment scale.

Trial	Suicides, no. of patients	Movement Disorders, no. of patients	Movement Disorders Assessment Scales, Mean (SD) change from baseline
**Kahn et al. 2021**	Successful = 0	Dyskinesia = 6	AIMS = − 0.1 (0.8)
	Ideation = 18	Parkinsonism = 11	SAS = − 01 (1.0)
	Behaviour = 1	Akathisia = 4	BARS = 0.0(0.4)
**Yagoda et al. 2021**	Successful = 0	Dyskinesia = 7	AIMS = 0.0 (0.81)
	Ideation = 5	Parkinsonism = 19	SAS = 0.1 (1.08)
	Behaviour = 1	Akathisia = 13	BARS = 0.0 (0.42)
**Potkin et al. 2020**	Successful = 0	Dyskinesia = 2	AIMS = 0.1 (0.8)
	Ideation = 0	Parkinsonism = 5	SAS = 0.0 (0.78)
	Behaviour = 0	Akathisia = 8	BARS = 0.0 (0.47)
**Correll et al. 2020**	Successful = 0	N/A	N/A
	Ideation = 0		
	Behaviour = 0		
**Martin et al. 2019**	Successful = 0	N/A	N/A
	Ideation = 0		
	Behaviour = 0		

N/A = Not Assessed, AIMS = Abnormal Involuntary Movement Scale, BARS= Barnes Akathisia Rating Scale, SAS= Simpson-Angus Scale, SD= Standard Deviation.

## 14 Movement disorders

In the phase III trial reported by Kahn et al. [41] mean (SD) changes in movement disorder assessments from baseline to week 52 were −0.1 (0.8) for the Abnormal Involuntary Movement Scale (AIMS) total score, 0.0 (0.4) for the Barnes Akathisia Rating Scale (BARS) global score, and −0.1 (1.0) for the Simpson-Angus Scale (SAS) total score. 6 (2.3%) patients had dyskinesia (defined as an AIMS score ≥3 on any of the first 7 items, or ≥ 2 on 2 or more of the first 7 items), while 11 (4.2%) patients experienced parkinsonism (defined as a SAS total score >3) and 4 (1.5%) patients had akathisia (defined as a BARS global score ≥2) [39]. Potkin et al. [31] reported that two patients in the OLZ/SAM group experienced dyskinesia. Change in AIMS total score was recorded as mean (SD) 0.1 (0.8) in OLZ/SAM group. Five patients experienced parkinsonism in the OLZ/SAM group, which was lower compared to the other treatment groups. There was a mean (SD) change of 0.0 (0.78) in SAS total scores for the OLZ/SAM group. Eight patients had akathisia with a mean (SD) change of 0.0 (0.47) in BARS total scores for the OLZ/SAM group [31]. In the phase III trial by Yagoda et al. [42] mean (SD) changes from baseline to week 52 in movement scale scores were recorded as 0.0 (0.81) for AIMS total, 0.0 (0.42) for BARS global, and 0.1 (1.08) for SAS score. Parkinsonism due to OLZ/SAM treatment occurred in 19 (6.9%) patients, dyskinesia in 7 (2.5%) patients, and akathisia in 13 (4.7%) patients [42].

## 15 Adverse events (AEs)

The adverse events due to OLZ/SAM therapy were displayed by almost all the RCTs included in this review. However, the severity of these events was different across the studies. For ease of understanding, these events are divided into further sub-headings below. All the results are shown for the OLZ/SAM group only.

### 15.1 The severity of AEs

AEs experienced by participants during trials were sub-categorized by authors into four main categories. These categories were mild AEs, moderate AEs, severe AEs, and discontinuation of treatment due to adverse events. We report this data in Table 3.

**Table 3:** Adverse events.

TRIALS	Severity of AEs	Nervous system disorders	Metabolism and nutritional disorders	Gastrointestinal disorders	Changes in blood chemistry	Serious AEs
R.S. Kahn et al.	Any = 161, Mild = 93, Moderate = 61, Severe = 7, Discontinuation = 15.	Headache.	Weight changes (increased and decreased), changes in creatine phosphokinase, changes in blood prolactin, changes in waist circumference.	Nausea.	Changes in blood glucose, insulin, HbA1C levels (non-linear), Changes in lipid profile, hypertension.	Extra drug doses, Suicidal ideation.
L.Sun et al.	Any = 16, Mild = 13, Moderate = 3, Severe = 0, Discontinuation = 2.	Not assessed	Weight changes (increased).	Dry mouth, constipation.	Changes in blood glucose, insulin, HbA1C levels	Suicidal ideation.
W.F. Martin et al.	Any = 127, Mild = 82, Moderate = 39, Severe = 6, Discontinuation = 21.	Somnolence, headache, dizziness, and sedation.	Weight changes (increased), changes in appetite (increased).	Dry mouth, constipation, Nausea.	Not assessed	Worsening of Schizophrenia,
Correll et al.	Any = 203, Mild = 106, Moderate = 87, Severe = 10, Discontinuation = 33.	Somnolence.	Weight changes (increased), changes in appetite (increased), changes in creatine phosphokinase, changes in waist circumference.	Dry mouth.	Changes in blood glucose, insulin, HbA1C levels (decreased), Changes in lipid profile	Worsening of Schizophrenia, Extra drug doses, Suicidal ideation.
S.G. Potkin et al.	Any = 73, Severe = 1, Discontinuation = 2.	Somnolence, headache, anxiety.	Weight changes (increased).	Dry mouth.	Not assessed	Worsening of Schizophrenia, Suicidal ideation.
S.Yagoda et al.	Any = 136, Severe = 8, Discontinuation = 15.	Somnolence, headache, and anxiety.	Weight changes (increased and decreased), changes in blood prolactin.	Dry mouth.	Changes in blood glucose, insulin, HbA1C levels (increased), Changes in lipid profile	Worsening of Schizophrenia, Extra drug doses, Suicidal ideation.
Lei Sun et al.	Any = 32, Severe = 0, Discontinuation = 4.	Somnolence, dizziness, and sedation.	Weight changes (increased and decreased).	Dry mouth, constipation, Nausea.	Not assessed	Worsening of Schizophrenia,
Burnette et al.	Any = 64, Mile = 31, Moderate = 27, Severe = 6, Discontinuation = 10	Headache.	Weight changes (increased).	Not assessed.	Changes in blood insulin (increased), hypertension.	Worsening of Schizophrenia, Suicidal ideation.

AE = Adverse Events.

Overall, the rate of AEs among participants was low, and it was found that treatment discontinuation during the trials was lesser in OLZ/SAM group compared to the olanzapine-only group [32]. The common reasons for discontinuation expressed in the trials were exacerbation of schizophrenia, high HbA1C, elevated hepatic enzyme levels and increased psychotic disorders.

### 15.2 Nervous system disorders

A number of nervous systems disorders were observed across the included studies. In phase III clinical trial, somnolence was recorded in 58 patients as one of the most commonly occurring adverse events [31]. While in another study 9 patients experienced somnolence [42]. Another study reported 23 patients who experienced somnolence and 11 patients who experienced headaches [42]. While Martin et al. also reported 29 patients experiencing somnolence and 5 experiencing headaches [33]. Potkin et al. reported somnolence in 12 patients and headache in 8 patients [31]. The phase II trial by Brunette et al. only had 2 patients who experienced a headache [43].

Amongst the less severe nervous system disorders, anxiety and dizziness were observed in two studies each. Potkin et al. [31] reported 8 patients with anxiety, while Yagoda et al. [42] reported 7 patients who experienced anxiety. In a separate trial, 4 patients had dizziness [44], while in another 9 patients were observed to have dizziness [33]. Only one trial mentioned sedation in 12 patients as an adverse event [33].

### 15.3 Metabolism and nutrition disorders

Weight change is the most significant side effect of the drug OLZ/SAM as it was reported by all the clinical trials included in this study. A trial by Kahn et al. [41] compared the changes in body weight amongst their incorporated population. The proportion of patients with decreased weight was examined to be higher (n = 23) than those with increased weight (n = 16) compared to the baseline values. Yagoda et al. [42] and Sun et al. [44] reported non-similar findings, the frequency of patients with increased weight was found to be higher in these studies than the patients with decreased weight as compared to the baseline values. Interestingly, in a multicenter phase II trial having patients with comorbid alcohol use disorder (AUD), the authors reported that a higher proportion of patients treated with OLZ/SAM experienced weight gain (n = 16). While 14 patients had an increase in weight when treated with olanzapine only [43]. Increased appetite was outlined by two studies as a complication observed among 30 and 16 participants of the OLZ/SAM group respectively [32,33]. Changes in the blood prolactin were also expressed in two studies [41,42]. Prolactin levels were found to be decreased in one study by −5.5(18.9) ng/mL in females and −0.9(6.4) ng/mL in males compared to baseline values [41]. Creatine phosphokinase was found to be increased in two studies [31,41]. Waist circumference was noticed to be increased in a trial by Correll et al. [32]. Another study reported no change in waist circumference among the patients relative to baseline [41].

### 15.4 Gastrointestinal disorders

Dry mouth is the most common AE under this category reported by six different studies. A study by Correll et al. reported 35 patients with dry mouth as a complication which is the highest number of cases amongst all studies [31]. Potkin et al. compared the side effect of mouth dryness among OLZ/SAM combination therapy and olanzapine monotherapy groups. The first-mentioned group was observed to have suffered more with the complications of mouth dryness (n = 10) as compared to the olanzapine monotherapy group (n = 7) [31]. Constipation was reported by Sun et al. [36] and Martin et al. [33] but a negligible number of participants faced it. Three studies documented nausea, amongst which one reported 14 patients who experienced it [33]. The remaining had an insignificant number of patients with the side effect [41,44].

### 15.5 Changes in the blood chemistry

Olanzapine/Samidorphan has a profound effect on blood glucose, insulin, and HbA1C which was reported by five trials. Changes in lipid profiles were examined by three trials. Correll et al. [24] their study measured the levels of glucose, HbA1C, insulin dosage, triglycerides, high density and low-density lipoproteins (HDL, LDL), and cholesterol during the 2nd, 4th, 8th, 12th, and 24th week of the trial. All the aforementioned blood levels were recorded to decrease linearly, relative to their baseline values. Furthermore, two studies calculated the fasting blood glucose, HbA1C, fasting insulin, fasting triglycerides, fasting HDL, fasting LDL, and fasting cholesterol levels during different weeks [41,42]. The values were found to be non-linearly fluctuating. Another trial by Brunette et al. [43] examined the increased blood insulin levels to be higher in OLZ/SAM combined regimen as compared to olanzapine monotherapy. Moreover, two studies reported cases of hypertension in 9 patients receiving the OLZ/SAM regimen [41,42].

### 15.6 Serious AEs

Worsening of Schizophrenia was reported in six studies. Yagoda et al. reported that 9 patients had worsening schizophrenia, which is the most number of cases among the six studies [42]. Potkin et al. [31] and Martin et al. [33] in each of their studies reported a single case of schizophrenia exacerbation. A phase III trial conducted in the US compared schizophrenia worsening between OLZ/SAM group and the olanzapine-only group; it was observed that the last-mentioned group showed more cases of schizophrenia exacerbation. Only one case of schizophrenia was reported by Sun et al. after OLZ/SAM treatment [36]. Three studies reported that the cases of serious adverse events occurred in patients who were administered with an extra dosage of OLZ/SAM regimen in order to control the previously diagnosed schizophrenia [32,41,42].

## 16 Discussion

The aim of our review is to overview the pharmacokinetics, pharmacodynamics, safety, efficacy and adverse events associated with the recently FDA approved combination of OLZ/SAM for the treatment of schizophrenia. Studies assessing the safety and efficacy of the drug recombination of samidorphan and olanzapine showed decrease in PANSS total scores and CGI-S scores corroborating that long term treatment with OLZ/SAM was linked with antipsychotic efficacy and was well tolerated by schizophrenic patients. Patients treated with the drug recombination not only showed improvements in the symptoms of schizophrenia as evident by the comparatively lower PANSS and CGI-S scores but also exhibited low rates of discontinuation in trials, validating that OLZ/SAM is well tolerated in patients with schizophrenia [32,33,41,42]. Upon monitoring, patients undergoing treatment with OLZ/SAM did not display any significant ECG changes confirming that OLZ/SAM does not affect ECG parameters and the QTC interval [44]. When patients were evaluated for metabolic changes, OLZ/SAM did not have any significant effect on lipid parameters and glycemic control was maintained in patients undergoing long term treatment with OLZ/SAM [32,41,42].

Various trials reveal that while OLZ/SAM leads to weight gain in schizophrenic patients, the weight gain is significantly less and stabilizes after a while as compared to olanzapine monotherapy since patients in the OLZ/SAM group demonstrated minimal changes in waist circumference as compared to patients in the olanzapine group [41,42]. Patients treated with OLZ/SAM also showed minimal changes from their baseline weight when compared to patients who were only administered olanzapine [32,33].

The efficacy of the combination regimen as compared to other drugs can be attributed to Samidorphan's ability to counter the weight gain linked with olanzapine administration by blocking adipose glucose uptake and by countering insulin resistance caused by olanzapine use as demonstrated in animal models [32]. While structurally similar to the opioid receptor antagonist naltrexone, samidorphan is more efficacious for treating schizophrenia due to its affinity being five times greater at mu-opioid receptors and its increased oral bioavailability as compared to naltrexone [45]. OLZ/SAM can also be considered safer as compared to other treatment options because it is administered in the form of a single tablet hence avoiding any issues that are encountered when taking additional medication with a prescribed tablet i.e side effects and drug-drug interaction impact [35]. While other treatment options are also being scrutinized for their ability to alleviate the weight gain associated with antipsychotics' use, so far no such evidence has come to light since the pharmacological options under assessment either have major adverse events associated with their use or lack efficacious results that could outweigh potential AEs [35].

Some of the adverse effects of the OLZ/SAM combination are distressing, namely the ideation of suicide by patients [32,41,42]. Further research is needed to fully understand the extent to which the patients are affected by suicidal thoughts so they can be monitored effectively according to the severity of their symptoms.

## 17 Strengths and limitations of review

To the author's best knowledge, this is the first review to report the combined efficacy and tolerance of OLZ/SAM drug. The review covers clinical trials with large sample sizes. Despite the significant findings, our review has certain limitations. The RCTs and clinical trials included have highlighted a significant number of patients who withdrew from the trials either due to lack of efficacy or adverse effects associated with OLZ/SAM, contributing to a high proportion of missing data. Out of the 8 trials included in our study, 3 showed a high risk of bias [33,42,43]. The risk of bias can be attributed to insufficient information regarding the randomization process or lack of it in the aforementioned studies. However, the interpretation of results has been made easy considering that the studies included in this review had a similar patient population and the dosage of OLZ/SAM was similar across the various trials.

## 18 Conclusion

The FDA-approved drug recombination of OLZ/SAM for the treatment of schizophrenia revealed efficacious outcomes and was generally well tolerated by patients partaking in various trials. While patients undergoing treatment with OLZ/SAM exhibited various adverse effects including substantial weight gain and in some cases suicidal ideation, the efficacy of the regimen in reducing the symptoms and severity of schizophrenia outweighs the risks. Further clinical trials are required to assess the long-term side effects of the regimen, however, the potential of samidorphan in mimicking the efficacy of olanzapine while mitigating olanzapine-induced weight gain makes it a promising agent for improving symptoms and health outcomes in schizophrenic patients.

## Ethical approval

Not required.

## Source of funding

None.

## Author contribution

Authors S.T.R, A.H.S, and Z.K conceived the idea for the review and coordinated the review process. Authors L.I, M.S, and A.A.S performed the literature search and data analysis for their assigned sections and drafted their assigned sections. S.T.R, A.H.S, and M.J.T created the first draft of the manuscript and developed the Figures and Tables in the review. M.S.A, M.J.T, Z.K, Z.J, and A.A provided critical review and inputs. All authors read, critically revised and approved the final manuscript.

## Trail registry number

None.

## Garantor

Muhammad Sohaib Asghar.

## Availability of data and material

All data generated were included within the manuscript and supplementary materials.

## Provenance and peer review

Externally peer reviewed, not commissioned.

## Declaration of competing interest

None.

## Footnotes

Appendix A Supplementary data to this article can be found online at https://doi.org/10.1016/j.amsu.2022.104115.
