# Supplementary material for: Samidorphan/olanzapine combination therapy for schizophrenia: Efficacy, tolerance and adverse outcomes of regimen, evidence-based review of clinical trials
Source: Ann Med Surg (Lond). 2022 Jun 30;79:104115. doi: 10.1016/j.amsu.2022.104115 (PMC9289510; doi:10.1016/j.amsu.2022.104115)
Supplement: Multimedia component 1 [file mmc1.docx]

Supplementary Table 1. Search history.

| Search database | Search term | Results |
| --- | --- | --- |
| Pubmed | ("olanzapine"[MeSH Terms] OR "olanzapine"[All Fields] OR "olanzapin"[All Fields] OR "olanzapine s"[All Fields]) AND ("3 carboxamido 4 hydroxynaltrexone"[Supplementary Concept] OR "3 carboxamido 4 hydroxynaltrexone"[All Fields] OR "samidorphan"[All Fields]) AND ("schizophrenia"[MeSH Terms] OR "schizophrenia"[All Fields] OR "schizophrenias"[All Fields] OR "schizophrenia s"[All Fields]) | 24 |
| Google Scholar | (olanzapine) AND (samidorphan) AND (schizophrenia) | 195 |
| Sciencedirect | (olanzapine) AND (samidorphan) AND (schizophrenia) | 45 |
| Ovid SP | (olanzapine) AND (samidorphan) AND (schizophrenia) | 2946 |
